# A Comparative Study of Stapler and Laser Hemorrhoidoplasty in the Treatment of Second-Degree and Third-Degree Hemorrhoids

**DOI:** 10.7759/cureus.87834

**Published:** 2025-07-13

**Authors:** Hemant Bhanarkar, Ashutosh D Jadhao, Bhupesh Tirpude, Vikrant Akulwar, Gayatri Deshpande, Raj Gajbhiye, Nikita Monteiro

**Affiliations:** 1 General Surgery, Government Medical College and Hospital Nagpur, Nagpur, IND

**Keywords:** 2nd and 3rd degree hemorrhoids, ano-rectal diseases, hemorrhoids, laser hemorrhoidoplasty, stapler hemorrhoidopexy

## Abstract

This study compares the effectiveness of stapled hemorrhoidopexy and laser hemorrhoidoplasty in treating second- and third-degree hemorrhoids. A total of 60 patients were included, divided equally between the two treatment groups. The average age of patients in the stapled hemorrhoidopexy group was 49.31 ± 11.26 (years), while in the laser hemorrhoidoplasty group, it was 52.59 ± 9.51 years. The procedures were conducted between August 4, 2022 and July 31, 2024, at a tertiary care hospital. Outcomes, such as postoperative pain, bleeding, recurrence, and recovery, were analyzed. Results showed that patients treated with laser hemorrhoidoplasty experienced significantly less postoperative pain, reduced blood loss, shorter hospital stays, faster recovery, and quicker return to daily activities, with all differences being statistically significant (P < 0.001). Additionally, the laser procedure had a shorter average surgery duration compared to the stapled method. The average duration of surgery was 30.17 ± 6.88 minutes in the stapler group compared to 24.48 ± 3.45 minutes in the laser group. Overall, laser hemorrhoidoplasty demonstrated superior efficacy and patient outcomes, making it a more favorable treatment option for managing grades II and III hemorrhoids.

## Introduction

Hemorrhoidal disease is the most common anorectal disorder worldwide, affecting a substantial portion of the population and posing notable medical and socioeconomic burdens. Its global prevalence varies widely, estimated between 2.9% and 27.9%, with symptoms reported in more than 4% of individuals [1]. The anorectal vascular cushions, in conjunction with the internal anal sphincter, are essential for continence, as they help maintain closure of the anal canal. Hemorrhoids are believed to develop due to the downward displacement of the suspensory muscle of Treitz, leading to discomfort and a noteworthy decline in the patient's quality of life [2,3].

Advancements in the understanding of the anatomy and pathophysiology of hemorrhoidal disease have paved the way for ongoing improvements in surgical techniques, with the goal of achieving effective treatment while minimizing tissue disruption and complications [4]. Contemporary procedures allow for the removal of hemorrhoids using less invasive approaches, significantly reducing recovery time and enabling patients to resume normal activities more quickly compared to older, more invasive methods involving extensive ligation. These innovations have led to better clinical outcomes, including less postoperative discomfort, quicker healing, and a lower incidence of both early and delayed complications [4,5]. Management strategies for symptomatic hemorrhoids have evolved and now include a range of options, from conservative medical therapies to non-surgical and surgical procedures. Minimally invasive, non-surgical techniques, such as rubber band ligation, sclerotherapy, cryotherapy, infrared coagulation, laser therapy, and diathermy, are typically performed in outpatient settings without anesthesia, and are generally recommended for treating grade I to III hemorrhoids [4,6,7].

Surgical treatment continues to be the most dependable option for managing hemorrhoids, particularly in severe cases such as irreducible (grade IV) piles or when conservative approaches fail to effectively control bleeding. Surgical intervention is often advised for patients with prominent external hemorrhoidal components, enlarged anal papillae, associated anal fissures, significant thrombosis, or persistent symptoms following multiple sessions of rubber band ligation. Common surgical procedures include stapled hemorrhoidopexy, utilizing various instruments such as scalpels, scissors, electrocautery devices, or laser technology [3,4].

Even with the progress in surgical approaches, several issues remain unresolved, including postoperative pain, discomfort, mucous discharge, limited ability to perform daily tasks, and the risk of recurrence. Although multiple surgical techniques are available, there is still no consensus on the most effective treatment strategy [8]. Conventional hemorrhoidectomy is known for its low recurrence rate but is frequently associated with considerable postoperative pain. In contrast, newer procedures generally result in less discomfort after surgery but may carry a higher likelihood of recurrence and introduce complications such as tenesmus or urgency-related incontinence [9-11]. Stapled hemorrhoidopexy uses a circular stapler to remove a ring of mucosal and submucosal tissue located above the dentate line [2].

Several studies have highlighted that stapled hemorrhoidopexy is associated with reduced pain, shorter hospital stays, and faster recovery when compared to traditional hemorrhoidectomy, contributing to its growing preference among patients [2,4,12]. Laser hemorrhoidoplasty, on the other hand, is a more recent advancement and a minimally invasive, virtually painless, single-day surgical option for treating symptomatic hemorrhoids [13]. It has gained popularity as a routine outpatient procedure for hemorrhoid management [14].

One technique uses Doppler-guided laser coagulation to block the arterial blood supply to hemorrhoidal tissue, while another involves delivering laser energy directly into the hemorrhoids, inducing fibrosis. This fibrotic process causes the hemorrhoidal mass to shrink and adhere to the wall of the anal canal, thereby preventing further prolapse [15]. Lasers, with their various wavelengths, offer distinct therapeutic benefits, as each wavelength interacts differently with tissue. Commonly used lasers include carbon dioxide lasers, argon lasers, and neodymium: yttrium-aluminum garnet (Nd:YAG) lasers [16]. Laser treatment helps minimize tissue trauma, promotes effective bleeding control, and can significantly reduce both surgery duration and hospital stay [15,16].

This study aimed to evaluate and compare the effectiveness of stapled hemorrhoidopexy and laser hemorrhoidoplasty in the treatment of second- and third-degree hemorrhoids.

## Materials and methods

This hospital-based interventional study was conducted over a two-year period, from August 4, 2022 to July 31, 2024, in the surgical outpatient department of a tertiary-care hospital in central India. It included patients with a confirmed diagnosis of grade II or III hemorrhoids, with or without accompanying fissures. These patients are typically presented with symptoms such as rectal bleeding, pain, and discomfort during defecation.

Informed consent was obtained from all participants, and the study received prior approval from the Institutional Research Ethics Committee. The inclusion criteria encompassed adults of all age groups diagnosed with grade II/III hemorrhoids, with or without fissures. Patients who chose not to participate or who were discharged against medical advice were excluded from the study. Additional exclusion criteria included thrombosed hemorrhoids, perianal hematoma, ulcerated hemorrhoids, anal strictures, proctitis, recto-sigmoid growths, as well as pregnant and lactating women.

All patients received a thorough clinical evaluation, accompanied by laboratory tests, including a complete blood count to assess hemoglobin levels in cases of bleeding hemorrhoids and determine the necessity for preoperative blood transfusion. Other tests conducted included liver function tests and prothrombin time analysis.

Patients were randomly assigned to two groups using the chit method: Group A received stapled hemorrhoidopexy, while Group B underwent laser hemorrhoidoplasty. Data collection followed a standardized proforma. Postoperative pain was evaluated using the Visual Analog Scale (VAS) at 12 and 24 hours after surgery. The risk of postoperative bleeding (PR bleed) was assessed based on the frequency, amount, and severity of bleeding episodes.

Patients were typically discharged on the first postoperative day and advised to return for a follow-up visit after one week, during which VAS scores were reassessed and any complications or concerns were documented. A second follow-up was scheduled at four weeks to evaluate for signs of anal stenosis, such as straining or difficulty during defecation.

Postoperative incontinence was evaluated using the Jorge-Wexner score, while recurrence was monitored through regular follow-ups in the outpatient department for up to three months.

In Group A, patients were positioned in the lithotomy posture, followed by a digital rectal examination. This was followed by anal dilation and manual repositioning of any prolapsed hemorrhoids or mucosal protrusions. The stapled hemorrhoidopexy procedure, following the technique introduced by Longo, was then conducted [17]. A 2/0 polypropylene purse-string suture was inserted approximately 4 cm above the dentate line, encircling the mucosal and submucosal layers. A circular stapling device (PPH 03, Ethicon Endo-Surgery™, USA) was then used to perform mucosal resection and fixation. The excised tissue was preserved for histopathological analysis. To conclude the procedure, an absorbable gelatin sponge dressing was inserted into the anal canal.

In Group B, after conducting a thorough physical examination and proctoscopy, laser therapy was administered using the Lasotronix system (Poland). Lasotronix Laser Machine for Laser Hemorrhoidoplasty is 15 Watt and of 1470 nm wavelength. Bare tip Laser fiber is used for the Laser hemorrhoidoplasty procedure in which around 200 Joules to 300 Joules energy is given in a Single pile mass. Patients were positioned in the lithotomy posture, and a disposable proctoscope with a 23 mm diameter was introduced into the anal canal. A 980 nm diode laser, connected to a 1,000-micron optical fiber, was used to deliver pulsed laser energy, carefully calibrated to avoid damage to adjacent healthy tissues. Tissue shrinkage was precisely controlled by adjusting the power output and duration of laser exposure. The fiber delivered five laser pulses, each lasting 1.2 seconds at 13 watts, with a 0.6-second pause between pulses, achieving approximately 5 mm of tissue coagulation depth.

A total of 60 patients participated in the study, evenly distributed into two groups, Group A and Group B, each consisting of 30 individuals. The sample size was calculated through a power analysis conducted using G*Power software version 3.0.1 (Franz Faul, University of Kiel, Germany). A p-value below 0.05 was regarded as indicative of meaningful statistical significance. The data were compiled using Microsoft Excel and subsequently analyzed with IBM SPSS Statistics for Windows, Version 24.0 (IBM Corp., USA), along with GraphPad Prism Version 8.4.2. Continuous variables were presented using mean and standard deviation, whereas categorical variables were described using counts and percentages. Statistical analysis for categorical data was done using the Chi-square test and for continuous data using independent t-test.

## Results

Tables 1 and 2 present a comparison of age and sex distribution between the stapler and laser groups, respectively. The average age of patients in the stapler group was 49.31 ± 11.26 years, while in the laser group it was 52.59 ± 9.51 years, with no statistically noteworthy difference observed between the two groups (P = 0.236). Regarding sex distribution, 23 (79.3%) of patients in the stapler group were male, while 24 (82.8%) of patients in the laser group were male, with females representing 6 (20.7%) and 5 (17.2%) in the stapler and laser groups, respectively. The difference in sex distribution between the two groups was also not statistically noteworthy as P equal to 0.738. These results indicate that both groups had similar age and sex profiles, with no significant differences in either parameter.

**Table 1:** Demographic variables (age)

Parameter	Group	Mean	SD	P-value
Age (years)	Stapler	49.31	11.26	0.236
	Laser	52.59	9.51	

**Table 2:** Demographic variables (sex) N: Number of patients

Parameter	Group	Males, N (%)	Females, N (%)	P-value
Sex	Stapler	23 (79.3%)	6 (20.7%)	0.738
	Laser	24 (82.8%)	5 (17.2%)	

Table 3 compares the incidence of various postoperative symptoms between the stapler and laser groups. Both groups showed identical rates of PR bleed and constipation, with 25 (86.2%) of patients experiencing PR bleed and 17 (58.6%) experiencing constipation in each group (P = 1.000 for both). Pain during defecation was reported by 1 (3.4%) of patients in the stapler group and 2 (6.9%) in the laser group, though this difference was not statistically noteworthy (P = 0.5). Regarding mass protruding from the rectum, 16 (55.2%) of patients in the stapler group and 15 (51.7%) in the laser group reported this symptom, with no noteworthy difference between the groups (P = 0.792). Fissures were reported by 1 (3.4%) of patients in the stapler group and 2 (6.9%) in the laser group, again with no significant difference (P = 0.5). Overall, the results indicate that both groups had similar postoperative symptoms, with no noteworthy differences observed in terms of bleeding, constipation, pain, mass protrusion, or fissures.

**Table 3:** Clinical symptoms PR bleed: Postoperative bleeding

Parameters		Stapler Group	Laser Group	P-value
PR bleed	Yes	25 (86.2%)	25 (86.2%)	1
	No	4 (13.8%)	4 (13.8%)	
Constipation	Yes	17 (58.6%)	17 (58.6%)	1
	No	12 (41.4%)	12 (41.4%)	
Pain during defecation	Yes	1 (3.4%)	2 (6.9%)	0.5
	No	28 (96.6%)	27 (93.1%)	
Mass protruding from rectum	Yes	16 (55.2%)	15 (51.7%)	0.792
	No	13 (44.8%)	14 (48.3%)	
Fissure	Yes	1 (3.4%)	2 (6.9%)	0.5
	No	28 (96.6%)	27 (93.1%)	

Table 4 compares the distribution of hemorrhoid grades and positions between the stapler and laser groups. In terms of hemorrhoid grade, both groups had similar proportions, with 13 (44.8%) of patients in the stapler group and 14 (48.3%) in the laser group presenting with Grade II hemorrhoids, and 16 (55.2%) of stapler group patients and 15 (51.7%) of laser group patients having Grade III hemorrhoids. The difference between the two groups was not noteworthy as the P-value was 0.792. Regarding the position of the hemorrhoids, the stapler and laser groups had similar distributions. Many patients in both groups had hemorrhoids at the 3 and 7 o’clock positions (12 (41.4%) in the stapler group and 13 (44.8%) in the laser group), followed by those at the 3, 7, and 11 o’clock positions (9 (31%) in the stapler group and 5 (17.2%) in the laser group). The differences in hemorrhoid position between the groups were also not statistically significant (P-values: 0.572-1.000). The results indicate that both groups had similar grades and positions of hemorrhoids, with no statistically significant differences noted between them.

**Table 4:** Features of hemorrhoids N: Number of patients

Parameters		Stapler group (N, %)	Laser group (N, %)	P-value
Grade of hemorrhoids	Grade II	13 (44.8%)	14 (48.3%)	0.792
	Grade III	16 (55.2%)	15 (51.7%)	
Position of hemorrhoids	7 and 11 o'clock	4 (13.8%)	7 (24.1)	0.572
	3, 7, and 11 o'clock	9 (31%)	5 (17.2%)	
	3 and 7 o'clock	12 (41.4%)	13 (44.8%)	
	3 and 11 o'clock	4 (13.8%)	4 (13.8%)	
	No	28 (96.6%)	27 (93.1%)	

Table 5 presents a comparative analysis between the stapler and laser groups on various postoperative parameters. The results indicate that the stapler group had significantly longer surgery duration (30.17 ± 6.88 minutes) compared to the laser group (24.48 ± 3.45 minutes, P < 0.001). Similarly, the stapler group had higher blood loss (10.17 ± 2.11 ml) compared to the laser group (5.34 ± 1.29 ml, P < 0.001). In terms of pain, the VAS scores at 24 hours, one week, one month, and three months were consistently higher in the stapler group, with statistically significant differences (P < 0.001) across all time points. Regarding recovery, the stapler group had a longer hospital stay (3.28 ± 0.45 days) and took longer to return to normal activity (4.24 ± 0.44 days) compared to the laser group, which had shorter stays (1.28 ± 0.45 days) and faster recovery (2.24 ± 0.44 days). These findings highlight the advantages of laser surgery in terms of reduced operative time, lower blood loss, less postoperative pain, shorter hospital stay, and quicker recovery.

**Table 5:** Comparison of parameters related to surgery VAS: Visual Analog Scale

Parameter	Group	Mean	SD	P-value
Duration of surgery (minutes)	Stapler	30.17	6.88	<0.001
	Laser	24.48	3.45	
Amount of blood loss (ml)	Stapler	10.17	2.11	<0.001
	Laser	5.34	1.29	
VAS score at 24 hours	Stapler	0.28	0.84	<0.001
	Laser	0.07	0.37	
VAS score at 1 week	Stapler	0.10	0.31	<0.001
	Laser	0.03	0.19	
VAS score at 1 month	Stapler	0.10	0.31	<0.001
	Laser	0.03	0.19	
VAS score at 3 months	Stapler	0.10	0.31	<0.001
	Laser	0.03	0.19	
Days of hospital stay (days)	Stapler	3.28	0.45	<0.001
	Laser	1.28	0.45	
Return to normal activity (days)	Stapler	4.24	0.44	<0.001
	Laser	2.24	0.44	

As per the results present regarding postoperative complications and recurrence in Table 6, the present study demonstrated that both stapler and laser procedures for hemorrhoid management are associated with low rates of PR bleed, recurrence, and complications, with no statistically noteworthy differences between the two groups. PR bleed occurred slightly more often in the stapler group (three (10.3%) compared to one (3.4%) in the laser group), but the difference was not noteworthy (P = 0.306). Recurrence was observed in two (6.9%) of patients in the stapler group and one (3.4%) in the laser group (P = 0.500).

**Table 6:** Postoperative complications and recurrence PR bleed: Postoperative bleeding, N: Number of patients

Parameter		Stapler Group (N, %)	Laser Group (N,%)	P-value
PR bleed	Yes	3 (10.3%)	1 (3.40%)	0.306
	No	26 (93.1%)	28 (96.6%)	
Recurrence	Yes	2 (6.9%)	1 (3.4%)	0.5
	No	27 (93.1%)	28 (96.6%)	
Postoperative complications	Nil	27 (93.1%)	26 (89.7%)	0.503
	Misfiring of stapler	1 (3.4%)	0 (0.0%)	
	Hematoma	1 (3.4%)	2 (6.9%)	
	Abscess	0 (0.0%)	1 (3.4%)	

Most patients in both groups experienced no postoperative complications (27 (93.1%) in stapler group vs. (26) 89.7% in laser group; P = 0.503). Minor complications such as misfiring of the stapler, hematoma, and abscess formation were rare and distributed similarly between the groups. Overall, both stapler and laser techniques appear safe and effective, with comparable outcomes in terms of bleeding, recurrence, and postoperative complications.

## Discussion

Hemorrhoids are a widespread condition, especially affecting individuals aged 45 to 65 [18]. This disorder involves the swelling and inflammation of the vascular cushions in the anal canal, leading to discomfort, pain, and complications that can severely impact a person’s quality of life. Symptoms such as bleeding, itching, and pain during bowel movements often necessitate medical intervention, particularly in cases of second- and third-degree hemorrhoids. Over time, treatment options have evolved, offering a variety of surgical techniques to address hemorrhoids of varying severity [19,20].

In this study, the comparison of age between the stapler and laser groups revealed that the mean age in the stapler group was 49.31 ± 11.26 years, while in the laser group it was 52.59 ± 9.51 years (Table 7). Table 8 contains information regarding sex distribution, 23 (79.3%) of patients in the stapler group were male, compared to 24 (82.8%) in the laser group. Females accounted for 6 (20.7%) in the stapler group and 5 (17.2%) in the laser group. Elhefny et al. reported a mean age of 43.4 ± 11.34 years in the stapler group and 44.23 ± 13.39 years in the laser group [8]. Their study found that 63.3% of the stapler group and 46.6% of the laser group were male, while 36.6% and 53.3%, respectively, were female [8]. Viswanath et al. observed that the mean age in the stapler group was 53.4 ± 11.7 years, compared to 50.9 ± 15.8 years in the laser group. Males made up 51.7% of the stapler group and 48.3% of the laser group, with females representing 42.9% and 57.1%, respectively [21].

**Table 7:** Comparison of mean age

Study	Mean age in stapler group	Mean age in laser group
Elhefny et al. [8]	43.4 ± 11.34 years	44.23 ± 13.39 years
Viswanath et al. [21]	53.4 ± 11.7 years	50.9 ± 15.8 years
Present study	49.31 ± 11.26 years	52.59 ± 9.51 years

**Table 8:** Gender wise distribution

Study	Stapler group		Laser group	
	Male	Female	Male	Female
Elhefny et al. [8]	63.30%	36.60%	46.60%	53.30%
Viswanath et al. [21]	51.70%	42.90%	48.30%	57.1%,
Present study	23 (79.30%)	6 (20.70%)	24 (82.80%)	5 (17.20%)

In the present study, the most common presenting symptoms in both the stapler and laser groups were rectal bleeding and constipation (Table 9). Rectal bleeding was observed in 25 (86.2%) of patients in both groups. Constipation was reported by 17 (58.6%) of patients in both groups. Pain during defecation was relatively uncommon, occurring in 1 (3.4%) of stapler group patients and 2 (6.9%) of laser group patients. Additionally, a protruding mass from the rectum was reported by 16 (55.2%) of patients in the stapler group and 15 (51.7%) in the laser group.

**Table 9:** Comparison of symptoms

Study	Stapler group	Laser group
Elhefny et al. [8]	Pain (73.3%); Bleeding (70%); Constipation (80%); Pruritus (33.3%)	Pain (60%); Bleeding (66.6%); Constipation (73.3%); Pruritus (30%)
Viswanath et al. [21]	Pain (91.7%); Bleeding (44.4%); Itching (30.1%)	Pain (94.4%); Bleeding (50%); Itching (19.4%)
Present study	Rectal bleed: 25 (86.2%); Constipation: 17 (58.6%); Pain during defecation: 1 (3.4%); Protruding mass from the rectum: 16 (55.2%)	Rectal bleed: 25 (86.2%); Constipation; 17 (58.6%), Pain during defecation: 2 (6.9%); Protruding mass from the rectum: 15 (51.7%)

When compared to previous studies , Elhefny et al. reported high incidences of pain (73.3%), bleeding (70%), and constipation (80%) in the stapler group, with slightly lower but still significant rates in the laser group pain (60%), bleeding (66.6%), and constipation (73.3%) [8]. Pruritus was also reported in both groups, at 33.3% in the stapler group and 30% in the laser group (Table 9).

Viswanath et al. observed even higher rates of pain in both groups, which were 91.7% in the stapler group and 94.4% in the laser group [21]. Bleeding was less common in their study compared to others, occurring in 44.4% of stapler and 50% of laser group patients. Itching was also noted, reported by 30.1% in the stapler group and 19.4% in the laser group.

Overall, while rectal bleeding and constipation remained the most common symptoms across studies, the incidence of pain during defecation was notably lower in the present study compared to earlier reports. This could reflect differences in disease severity, patient demographics, or variations in clinical evaluation and symptom reporting

In the present study, Grade II hemorrhoids were observed in 13 (44.8%) of patients in the stapler group and 14 (48.3%) of patients in the laser group (Table 10). This indicates that nearly half of the patients undergoing either procedure were diagnosed with Grade II hemorrhoids.

When compared to previous studies, Elhefny et al. reported slightly lower rates, with 30% of patients in the stapler group and 36.6% in the laser group presenting with Grade II hemorrhoids [8]. Similarly, Viswanath et al. documented 40.9% of stapler group patients and a higher 59.1% of laser group patients with Grade II disease [21].

Overall, the findings from the present study are consistent with earlier reports, showing a significant proportion of Grade II hemorrhoids among patients treated with both stapler and laser techniques. However, the present study shows a slightly higher prevalence of Grade II cases in the stapler group compared to Elhefny et al. and Viswanath et al., suggesting potential variations in patient selection or disease severity across different study populations [8,21].

**Table 10:** Commonest grade of hemorrhoids

Study	Stapler group	Laser group
Elhefny et al. [8]	Grade II hemorrhoids (30%)	Grade II hemorrhoids (36.6%)
Viswanath et al. [21]	Grade II hemorrhoids (40.9%)	Grade II hemorrhoids (59.1%)
Present study	Grade II hemorrhoids 13 (44.8%)	Grade II hemorrhoids 14 (48.3%)

In the present study, the mean operative time was significantly longer in the stapler group (30.17 ± 6.88 minutes) compared to the laser group (24.48 ± 3.45 minutes), with a P-value of less than 0.001, indicating a statistically noteworthy difference (Table 11). These findings are in line with those reported by Elhefny et al., where the stapler group had a mean operative time of 37 ± 4.46 minutes, significantly longer than the laser group at 25.1 ± 2.96 minutes (P < 0.001) [8]. Similarly, Kaushal et al., observed shorter operative times with the laser technique (24.6 minutes) compared to the stapler method (28.6 minutes), though the P-value was greater than 0.001, suggesting the difference was not noteworthy in their study [4].

**Table 11:** Mean operative time and blood loss

Mean operative time			
Study	Stapler group	Laser group	P-value
Elhefny et al. [8]	37 ± 4.46 minutes	25.1 ± 2.96 minutes	< 0.001
Kaushal et al. [4]	28.6 minutes	24.6 minutes	> 0.001
Present study	30.17 ± 6.88 minutes	24.48 ± 3.45 minutes	< 0.001
Mean blood loss			
Kaushal et al. [4]	11.64 ml	8.32 ml	0.011
Present study	10.17 ± 2.11 ml	5.34 ± 1.29 ml	< 0.001

Regarding intraoperative blood loss, the present study showed that the stapler group had a mean blood loss of 10.17 ± 2.11 ml, significantly higher than the laser group, which had a mean blood loss of 5.34 ± 1.29 ml (P < 0.001). These results align with those of Kaushal et al., who reported mean blood losses of 11.64 ml in the stapler group and 8.32 ml in the laser group, with a noteworthy difference (P = 0.011) [4].

Overall, these findings consistently suggest that the laser procedure is associated with shorter operative times and reduced blood loss compared to the stapler technique. This may be attributed to the minimally invasive nature of the laser method, leading to lesser tissue trauma and more efficient hemostasis.

In the present study, the mean postoperative pain score was identical in both groups, with a value of 0.10 ± 0.31 for both the stapler and laser groups (Table 12). Despite the same mean value, the P-value was less than 0.001, indicating a notified difference, due to the low variability and small SD in pain scores.

**Table 12:** Postoperative pain scores

Study	Stapler group	Laser group	P-value
Elhefny et al. [8]	1 ± 0.38	0.38 ± 0.49	< 0.001
Kaushal et al. [4]	3.6	1.88	> 0.001
Present study	0.10 ± 0.31	0.10 ± 0.31	< 0.001

When compared with previous studies, Elhefny et al. reported higher postoperative pain scores, with the stapler group showing a mean pain score of 1 ± 0.38 and the laser group 0.38 ± 0.49, a difference that was also statistically significant (P < 0.001) [8]. Kaushal et al. found even higher pain scores in both groups, with 3.6 in the stapler group and 1.88 in the laser group, although the P-value was more than 0.001, indicating the difference was not statistically noteworthy in their study [4].

Overall, these findings suggest that the postoperative pain in laser technique is lower compared to the stapler method. The present study shows remarkably lower pain scores in both groups compared to reports presented by Kaushal et al. and Elhefny et al. [4,8]. This reflect improvements of the present study in surgical technique, anesthesia protocols, or postoperative pain management strategies.

In the present study, the hospital stay was significantly longer in the stapler group, which was 3.28 ± 0.45 days as compared to the laser group which was around 1.28 ± 0.45 days, with a P-value of less than 0.001, indicating a highly noteworthy difference (Table 13). This suggests that patients undergoing laser treatment had a faster recovery and earlier discharge compared to those treated with the stapler technique.

**Table 13:** Mean hospital stay

Study	Stapler group	Laser group	P-value
Elhefny et al. [8]	30.3 ± 8.43 hours	14.53 ± 5.6 hours	< 0.001
Kaushal et al. [4]	32.64 hours	21.44 hours	0.007
Present study	3.28 ± 0.45 days	1.28 ± 0.45 days	< 0.001

As per findings by Elhefny et al., the stapler group had a mean stay of 30.3 ± 8.43 hours, which is significantly longer than the 14.53 ± 5.6 hours recorded for the laser group (P < 0.001) [8]. Similarly, Kaushal et al. observed a longer hospital stay in the stapler group is 32.64 hours as compared to the laser group which is 21.44 hours, with a P value of 0.007 [4].

Overall, the laser procedure consistently results in a shorter hospital stay across studies, likely due to less postoperative pain, minimal tissue trauma, and faster recovery associated with laser surgery. These outcomes reinforce the advantages of laser hemorrhoidoplasty in terms of enhancing patient comfort and reducing hospitalization time.

In the present study, postoperative complications were minimal in both groups (Table 14). In the stapler group, 27 cases (93.1%) had no complications, while one (3.4%) experienced hematoma and one (3.4%) had misfiring of the stapler device. In the laser group, 26 patients (89.7%) had no complications, and two (6.9%) developed an abscess. The difference between the two groups was not statistically noteworthy (P = 0.503).

**Table 14:** Postoperative complications

Study	Stapler group	Laser group	P-value
Elhefny et al. [8]	Postoperative anal incontinence (10%)	Postoperative anal incontinence (0%)	-
Vishwanathan et al. [21]	Complications present (30.6%)	Complications present (44.4%)	0.224
Present study	No complications: 27 (93.1%); Hematoma: 1 (3.4%); Misfiring of the stapler: 1 (3.4%)	No complications: 26 (89.7%); Abscess formation: 2 (6.9%)	0.503

Comparing these findings with previous studies, Elhefny et al. reported postoperative anal incontinence in 10% of stapler group patients, whereas no cases of anal incontinence were noted in the laser group [8]. Vishwanathan et al. found overall complication rates of 30.6% in the stapler group and 44.4% in the laser group [21]. However, based on the P-value of 0.224, the results appear to be somewhat comparatively similar.

Overall, the present study showed a lower complication rate compared to earlier studies, with both surgical techniques demonstrating a good safety profile. Although minor complications like hematoma and abscess formation were observed, serious complications such as anal incontinence were absent in both groups, highlighting the overall effectiveness and safety of both stapler and laser techniques.

Limitations of the study

The study has several limitations that should be acknowledged. Firstly, the relatively small sample size restricts the generalizability of the findings, and larger-scale studies are required to validate these results and draw more definitive conclusions. Additionally, follow-up evaluations were only conducted one week and four weeks post procedure, which limits the ability to assess the long-term outcomes of laser hemorrhoidoplasty and stapler hemorrhoidopexy. Furthermore, the use of analgesics by patients after discharge was not monitored, potentially affecting the accuracy of the reported pain scores, especially at the one-week follow-up or later. These limitations highlight the need for further research with broader sample sizes, extended follow-up periods, and more rigorous post-discharge monitoring.

## Conclusions

This study provides the comparative effectiveness of stapler hemorrhoidopexy and laser hemorrhoidoplasty for treating second- and third-degree hemorrhoids. Our findings demonstrate that both techniques effectively alleviate symptoms and enhance patients' quality of life. However, each method has distinct advantages and considerations that can influence clinical decisions. Laser hemorrhoidoplasty, in particular, showed promising results, including reduction in blood loss, surgical duration, and hospital stays; faster recovery; and lower postoperative pain levels, even at the three-month follow-up. These benefits position laser treatment as a potentially less invasive alternative to stapler hemorrhoidopexy for managing second- and third-degree hemorrhoids. The study highlights the need for further research to assess long-term outcomes and recurrence rates, which will help optimize treatment protocols and improve patient education. Ultimately, this research aims to contribute to more personalized and effective care for individuals with hemorrhoidal disease.
